# Hybrid Setting for Minimally Invasive Mitral Surgery in Patients With Inferior Vena Caval Filters

**DOI:** 10.1016/j.atssr.2024.04.030

**Published:** 2024-05-24

**Authors:** Antonio Spitaleri, Cristina Barbero, Barbara Parrella, Giovanni Marchetto, Stefano Salizzoni, Michele William La Torre, Mauro Rinaldi, Marco Pocar

**Affiliations:** 1 Division of Cardiac Surgery, City of Health and Science (Città della Salute e della Scienza) and Department of Surgical Sciences, University of Turin, Turin, Italy; 2 Division of Cardiac Surgery, Scientific Institute for Research, Hospitalization, and Health Care Foundation (Fondazione IRCCS) San Gerardo dei Tintori, Monza, Italy; 3 Department of Clinical Sciences and Community Health, University of Milan, Italy

## Abstract

Current cardiac surgery has evolved to include hybrid and minimally invasive settings. In parallel, less invasive techniques have been extended to complex clinical scenarios and may prove even more beneficial in higher-risk patients. However, comorbidities and challenging anatomy still represent limitations to widespread application of this philosophy. Previously implanted filters in the inferior vena cava may limit the feasibility and safety of peripheral cannulation techniques. Successful minimally invasive operations in a hybrid setting in 2 patients with caval filters are reported.

Current cardiac surgery has evolved to include hybrid and minimally invasive strategies,^1^ whereas indications for mitral valve operations have progressively been extended to patients with increasing risk profiles, including advanced age, multiple reoperations, and comorbidities.^2^, ^3^, ^4^ Less invasive techniques are also more commonly performed in complex clinical scenarios, especially at high-volume tertiary care centers, and these techniques may prove even more beneficial in higher-risk and frail patients.^1^, ^2^, ^3^, ^4^ However, comorbidities and specific anatomic conditions, such as severe calcification or extensive infection, still represent major limitations to widespread application of this philosophy in routine daily practice. In this respect, the presence of a previously implanted filter in the inferior vena cava to prevent recurrent pulmonary embolism may limit the feasibility and safety of peripheral cannulation techniques and may be viewed as a contraindication for less invasive approaches. We report successful minimally invasive operations in a hybrid setting in 2 patients with caval filters.

## Case Reports

### Patient 1

**Figure 1:**
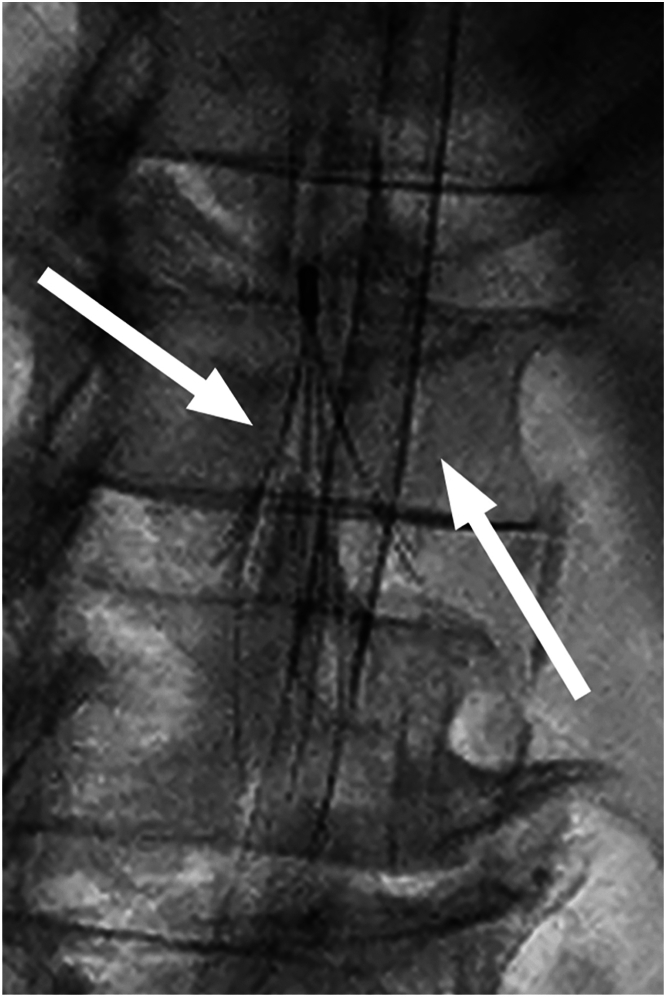
Fluoroscopic image of the transfemoral venous cannula crossing the struts of an ALN filter (ALN Implants Chirurgicaux) in the infrarenal vena cava (arrows).

A 72-year-old man with a complex coagulation disorder (antinuclear antibodies on Hep-2 cells, hyperhomocysteinemia, protein C and protein S anticoagulant deficiency, von Willebrand factor excess, and homozygous factor V VH1299R mutation) received a diagnosis of bilateral pulmonary embolism. He underwent ALN caval filter (ALN Implants Chirurgicaux) implantation and subsequent bilateral pulmonary endarterectomy with cardiopulmonary bypass, profound hypothermia and intermittent circulatory arrest. Severe mitral regurgitation with P2 flail was diagnosed after 7 years. Minimally invasive open heart surgery was judged not to be feasible because of the supposed impossibility of safe peripheral cannulation, and transapical off-pump mitral repair with neochordal (NeoChord, Inc) implantation was successfully performed. However, ventricular reverse remodeling led to neochordal detensioning, and moderate regurgitation relapsed after 1 year. Three years thereafter, mitral insufficiency progressed to severe, and the valve was rerepaired through a right minithoracotomy. Moderately hypothermic cardiopulmonary bypass was established in the hybrid operating room with bicaval peripheral jugular and femoral venous cannulation with a Seldinger technique. Inferior caval cannulation was guided fluoroscopically, and a 20-F cannula was inserted in the right atrium crossing the struts of the filter with no resistance and no displacement or deformation after retrieving the cannula, as confirmed fluoroscopically (Figure 1).

### Patient 2

A 71-year-old woman, also with a prothrombotic state (hyperhomocysteinemia, prothrombin g20210a and MTHFR c677t mutations), presented with deep vein thrombosis complicated by extensive bilateral pulmonary embolism. She subsequently underwent Celect Platinum caval filter (Cook) implantation, followed by bilateral endarterectomy for pulmonary hypertension and refractory cardiorespiratory failure. Severe mitral regurgitation with anterior leaflet prolapse and posterior leaflet retraction was identified after 8 years. It was treated uneventfully with minimally invasive valve replacement and associated left atrial appendage exclusion, with the same hybrid setting applied to the first case, with the use of a 20-F transfemoral inferior caval cannula.

## Comment

The boundaries of cardiac surgery have expanded to treat frail patients or challenging situations,^1^, ^2^, ^3^ often considered contraindications to minimally invasive approaches. Concerns also pertain to cannulation of peripheral vessels for cardiopulmonary bypass.^4^ Conversely, higher-risk patients are likely to benefit from the avoidance of primary or, even more, redo sternotomy.

Previous extensive dissection of the proximal pulmonary arteries likely increased technical hazards during reoperation. Conversely, mitral lesions were not amenable to transcatheter edge-to-edge repair. Our preferred approach comprises minimally invasive mitral valve surgery through a right minithoracotomy and, in redo cases, endovascular balloon occlusion rather than transthoracic aortic clamping. Similarly, endovascular balloon occluders have been used to avoid dangerous dissection for caval snaring.

**Figure 2:**
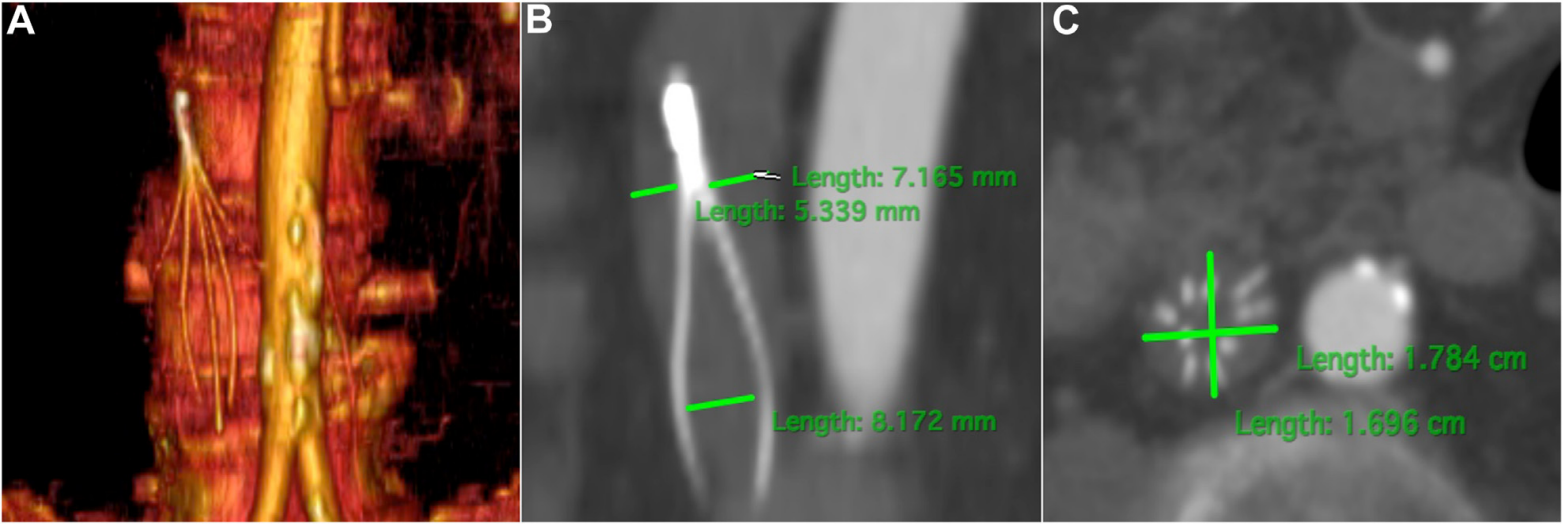
(A) 3-dimensional, (B) coronal, and (C) axial computed tomographic views of a filter implanted in the inferior vena cava.

We initially believed that previously implanted filters in the infrarenal vena cava would preclude safe transfemoral cannulation. However, the heart team opted for a minimally invasive approach as the safest strategy, mandating vascular assessment with total body computed tomography (CT) (Figure 2), and with the operations scheduled in the hybrid operating room to guide the procedure and address potential complications with the aid of additional fluoroscopy. After examination of the filter structure with CT, the distance between the metal struts would allow correct cannula positioning. We advocate for routine combined femoral and jugular venous cannulation in minithoracotomy mitral operations. This technique allows the placement of smaller cannulas for inferior caval drainage, an undoubted advantage in the presence of a caval filter. Vacuum-assisted drainage coupled with moderate hypothermia (28-30 °C core temperature) and consequently lower pump flow rates are also helpful, including in these particular cases. Besides, even though 17-F cannulas were adequate for transjugular superior caval drainage in our patients, larger cannulas were available.

Filter design rendered this approach possible, although this may not always be the case in relation to the space between the struts.^5^ Several filters are also retrievable.^6^ Additional maneuvers (ie, filter explantation, more peripheral cannula placement or transthoracic cannulation) at the expense of suboptimal drainage, more cumbersome operations, or wider thoracotomy incisions were unnecessary. The cannula crossed the filter uneventfully with no displacement or deformation, and decannulation was smooth. Another option is to perform the mitral surgery with induced ventricular fibrillation and no cardioplegic arrest, but this approach has inherent hazards of air trapping and embolism, coupled with longer cardiopulmonary bypass time to reduce flow at lower temperatures.

Despite no absolute contraindications for oral anticoagulation, we opted not to retrieve the devices, primarily because of baseline risk and a previous severe thromboembolic history in both patients. There was no evidence of pulmonary emboli after mitral surgery, a finding suggesting correct function of the filters, as confirmed by follow-up CT. Although modern hybrid operating rooms may also include CT,^7^ this was unnecessary in our experience. Conversely, the pivotal role of preoperative high-resolution CT evaluation cannot be overemphasized. Finally, minimally invasive valve surgery may also be considered in the presence of filters with narrower spaces between struts and thus deemed not to be compatible with a cannula crossing the filter itself. Adequate drainage may eventually be provided solely through the jugular route with a double-lumen cannula, such as the Protek Duo (CardiacAssist Inc), designed for right atrial–to–pulmonary artery circulatory support. In this setting, both lumina serve for drainage and as an additional venting line.^8^ This strategy also requires a hybrid operating room.

These 2 cases highlight the feasibility and safety of minimally invasive mitral surgery with standard transfemoral venous cannulation to cross a caval filter in selected patients. Filter design, high-resolution imaging, and a hybrid setting are keys to plan operations successfully and minimize surgical risks.
